# Partners in crime: The feedback loop between metabolic reprogramming and immune checkpoints in the tumor microenvironment

**DOI:** 10.3389/fonc.2022.1101503

**Published:** 2023-01-12

**Authors:** Jesus J. Benito-Lopez, Mario Marroquin-Muciño, Mario Perez-Medina, Rodolfo Chavez-Dominguez, Dolores Aguilar-Cazares, Miriam Galicia-Velasco, Jose S. Lopez-Gonzalez

**Affiliations:** ^1^ Laboratorio de Investigacion en Cancer Pulmonar, Departamento de Enfermedades Cronico-Degenerativas, Instituto Nacional de Enfermedades Respiratorias “Ismael Cosio Villegas”, Mexico City, Mexico; ^2^ Posgrado en Ciencias Biologicas, Universidad Nacional Autonoma de Mexico, Mexico City, Mexico; ^3^ Laboratorio de Quimioterapia Experimental, Departamento de Bioquimica, Escuela Nacional de Ciencias Biologicas, Instituto Politecnico Nacional, Mexico City, Mexico

**Keywords:** tumor microenvironment, metabolic reprogramming, immune cells, tumor cells, glucose and lactate, exogen amino acids, immune checkpoints

## Abstract

The tumor microenvironment (TME) is a complex and constantly changing cellular system composed of heterogeneous populations of tumor cells and non-transformed stromal cells, such as stem cells, fibroblasts, endothelial cells, pericytes, adipocytes, and innate and adaptive immune cells. Tumor, stromal, and immune cells consume available nutrients to sustain their proliferation and effector functions and, as a result of their metabolism, produce a wide array of by-products that gradually alter the composition of the milieu. The resulting depletion of essential nutrients and enrichment of by-products work together with other features of the hostile TME to inhibit the antitumor functions of immune cells and skew their phenotype to promote tumor progression. This review briefly describes the participation of the innate and adaptive immune cells in recognizing and eliminating tumor cells and how the gradual metabolic changes in the TME alter their antitumor functions. In addition, we discuss the overexpression of the immune checkpoints and their ligands as a result of nutrient deprivation and by-products accumulation, as well as the amplification of the metabolic alterations induced by the immune checkpoints, which creates an immunosuppressive feedback loop in the TME. Finally, the combination of metabolic and immune checkpoint inhibitors as a potential strategy to treat cancer and enhance the outcome of patients is highlighted.

## Introduction

The tumor microenvironment (TME) is composed of cellular and non-cellular elements that establish a dynamic interaction in which the concentration of nutrients and metabolic by-products constantly fluctuates over time, modifying the nutritional status of the cells. From a perspective of energy and biomass production, tumor cells show remarkable plasticity to adapt to the changing conditions of the TME, which allows them to overcome the diminished availability of oxygen and other nutrients. As a part of metabolic reprogramming, tumor cells may display alterations in their glycolytic activity, glutaminolysis, Fatty Acid Oxidation (FAO), Oxidative Phosphorylation (OXPHOS), etc (1). Aerobic glycolysis, also known as the Warburg effect, is probably the most evident and widespread metabolic adaptation in tumor cells. This process consists of a massive increase in glucose consumption and lactate production, even in the presence of oxygen, which provides tumor cells with a mechanism to fulfill the biosynthetic requirements to maintain uncontrolled proliferation. The increase in glucose consumption is concerted by changes in oxidative metabolism, activation of oncogenes, and inactivation of tumor-suppressor genes (2).

Nevertheless, studies on carbon flux have demonstrated that metabolic reprogramming on tumor cells is not limited to increased glucose uptake. Tumor cells employ multiple biosynthetic pathways to support their high proliferative rate, such as FAO or glutaminolysis, as well as increase the uptake of exogenous nutrients, such as amino acids like glutamine, tryptophan, and arginine. The depletion of these nutrients by consumption concomitantly promotes the accumulation of other by-products such as kynurenines and adenosine (3). Glutamine is an essential fuel for the increased demand of ATP, biosynthetic precursors, and reducing agents in cells with high proliferative rates, such as tumor and activated T cells. Glutaminolysis begins when glutamine enters the cell through the transporter ASCT2/SLC1A5 and is metabolized to glutamate and ammonium by the glutaminase enzyme (GLS). The resulting glutamate plays a key role in biomass production, redox homeostasis, and modulation of signaling pathways (4). In addition, the regulation of tryptophan concentration is essential for maintaining tissue homeostasis since the metabolism of this amino acid is related to nutrient sensing and metabolic response to cellular stress. In the TME, increased tryptophan metabolism by tumor cells induces the suppression of T cell responses (5). In multiple tumors, the overexpression of the enzyme that catabolizes tryptophan into kynurenines, Indoleamine-2,3-dioxygenase (IDO) has been reported to signal through the aryl hydrocarbon receptor (AhR) and to have immunosuppressive activity (6). Similarly, arginine availability in the TME plays a critical role in tumor cell proliferation and progression. Arginine is mainly synthetized from citrulline by the enzymes arginosuccinate synthase (AS) and arginosuccinate lyase (ASL) in a process known as the citrulline-nitric oxide cycle. This cycle represents the main arginine source for nitric oxide (NO) synthesis in immune cells (7). Arginine starvation assays have demonstrated that this amino acid is indispensable for tumor cells *in vitro* (8).

In the TME, tumor and stromal cells compete with immune cells for the uptake of these nutrients while producing a wide array of metabolic by-products with immunosuppressive activity. In nascent tumors, immune cells are immersed in an environment rich in nutrients that allows them to control tumor growth by eliminating susceptible tumor cells (9). Nevertheless, as tumors progress and the equilibrium phase of the immunoediting is established, metabolic alterations of tumor and stromal cells promote a metabolically hostile environment for effector immune cells. The resulting changes in concentrations of nutrients and by-products signal immune cells to end their antitumor functions and skew their phenotypes to enable immune escape and favor tumor cell proliferation. In this review, the participation of the innate and adaptive immune cells in recognizing and eliminating neoplastic cells is described. In addition, we discuss the main effects of the changes in the concentration of glucose, and some amino acids, as well as their by-products, on immune cells, highlighting the shifting of immune response from antitumor to tumor-promoting activity. Special focus is given to the crosstalk between immune checkpoints (ICs) and metabolic reprogramming in the establishment of an immunosuppressive feedback loop in the TME. Finally, current advances regarding therapy combining both metabolic and IC inhibitors (ICIs) are reviewed.

## Participation of the immune response in tumor development

The involvement of the immune system in the context of cancer has gained relevance in recent years since immune cells have been shown to play a dichotomic role in tumor development. On the one hand, the immunoediting theory postulates that immune cells recognize and eliminate susceptible tumor cells and that this process shapes tumor biology. On the other hand, it has been shown that chronic inflammation caused by an incomplete resolution process favors tumor progression. Excellent reviews have been published on the dual role of the immune system in tumor biology (10–12).

Although immune elimination of tumor cells mainly relies on the cytotoxic activity of CD8+ T cells, the activation of an effective antitumoral immune response involves the coordination of diverse innate and adaptive immune cells in a process regarded as the Cancer-Immunity Cycle (13). In nascent tumors, susceptible tumor cells are eliminated by innate immune cells. In this process, NK and NKT cells exert their cytotoxic function by releasing molecules with cytolytic potential, while phagocytic cells, by producing TNF-α and reactive oxygen (ROS) and nitrogen (RNS) species. Tumor cell death causes the release of tumor antigens and damage-associated molecular patterns (DAMPs) that stimulate the phagocytosis of dying tumor cells and promote the chemotaxis of immune cells from blood vessels. Moreover, the release of tumor antigens may also occur through the secretion of extracellular vesicles (EVs) by viable tumor cells. As previously reported, EVs contain tumor antigens and critical components that trigger the antitumor immune response (14). Tumor antigens released or contained in EVs are phagocyted by immature resident or arriving dendritic cells (DCs), processed through the ubiquitin-proteasome pathway, and resulting peptides are associated to class I and class II MHC molecules. Subsequently, DCs mature and migrate to draining lymph nodes to activate naïve CD4+ and CD8+ T cells. Activation triggers metabolic changes in T cells, such as aerobic glycolysis, necessary to sustain their proliferation and production of cytokines and cytotoxic molecules (15). Finally, T cells migrate through the bloodstream to infiltrate the tumor site, where effector CD8+ T cells (CTLs) recognize and eliminate tumor cells. This event promotes the release of more DAMPs and tumor antigens, perpetuating the Cancer-Immunity Cycle to eliminate susceptible tumor cells.

However, several reports indicate that tumors in advanced stages develop multiple mechanisms to evade immune destruction (16, 17). Among these mechanisms, reports highlight the diminishing of tumor cell antigenicity, recruitment of cells with regulatory activity and pro-tumoral function such as regulatory T cells (Tregs), M2 macrophages, and myeloid-derived suppressor cells (MDSCs), and the overexpression of molecules that limit the antitumor functions of immune cells, known as Immune Checkpoints (ICs) and their ligands. Recently, metabolic alterations in the TME promoted by tumor and stromal cells have been pointed out as an additional mechanism to evade immune destruction (18).

## Metabolic reprogramming in the TME

Multiple studies have demonstrated that cell metabolism is a housekeeping process to maintain cell survival, as well as a powerful guiding force that directs cell fate. As a result of the dynamic interplay between highly proliferating immune and tumor cells, local levels of nutrients such as glucose, glutamine, tryptophan, and arginine, among others are reduced in the TME. At the same time, metabolic by-products such as lactate, kynurenines, polyamines, and adenosine are accumulated. These metabolic alterations gradually change the environment of the TME, as illustrated by pH acidification of the milieu due to lactate accumulation, and act as a selective pressure that modifies the phenotypic features of the cellular components of the TME (19). Acidosis inhibits the glycolytic metabolism, thus affecting the proliferation of immune and some tumor cells.

As mentioned above, tumor cells can adapt to the changing conditions of the TME, such as hypoxia, deficiency of nutrients, iron accumulation, or acidic pH. For instance, some clones may express resistance mechanisms such as acid extruders (e.g., Na^+^, HCO3^-^ cotransporters, H^+^ ATPases, and Na^+^/H^+^ exchangers) to overcome and even take advantage of high lactate concentrations (20). Moreover, metabolic alterations in the TME promote tumor heterogeneity. Adverse features such as hypoxia and acidic pH induce cell cycle arrest or cell death in some tumor cell clones, while other clones reprogram their metabolism and acquire adaptation mechanisms that allow them to survive and proliferate.

Additionally, lactate is transported into cells through monocarboxylate transporters (MCTs) and signal through the specific G protein-coupled receptor 81 (GPR81), which has been reported to participate in cancer development and regulation of antitumor immune responses. Internalized lactate plays essential roles in tumor biology, such as improving tumor cell glycolysis by enhancing c-Myc signaling and the expression of the pyruvate kinase isoenzyme M2 (PKM2) (21, 22). As a result of lactate accumulation, emerging epigenetic modifications such as histone lactylation have been identified as important metabolic stress-related modifications that promote tumorigenesis (23, 24). However, immune cells lack the capacity to display these adaptation mechanisms, and metabolic alterations significantly affect their phenotype and effector mechanisms. For the correct elimination of tumor cells, CD4+ and CD8+ tumor-infiltrating lymphocytes (TILs), NK cells, and M1 macrophages rely on a highly glycolytic metabolism and amino acid consumption, while pro-tumoral immune cells, such as Tregs, M2 macrophages, and MDSCs mainly display an oxidative metabolism. Thus, changes in nutrient availability and concentration of metabolites in the TME impair the effector mechanisms of antitumor immune cells and favors the accumulation, survival, and suppressive functions of protumor immune cells.

In the next sections, we summarize the effects of the most common metabolic changes in glucose/lactate, glutamine, tryptophan/kynurenines, arginine, and adenosine, on inhibiting the tumor-infiltrating immune cells and their influence on the expression of the ICs and their ligands.

### Glucose and lactate

The introduction of high throughput technologies has confirmed Otto Warburg’s early observations that tumors consume more glucose than normal tissues and deepened the understanding of the biological role of glucose and lactate in the TME. In this regard, single-cell RNA-sequencing (scRNAseq) data from breast cancer patients showed that tumor cells display a gene signature associated with higher glucose consumption compared to normal epithelial cells (25), and glucose consumption by pancreatic ductal adenocarcinoma (PDAC) cell lines has been reported to impair proliferation of CD8+ T cells (26). Lactate, once considered a mere waste product of glycolytic metabolism, is now well known to play a critical role in establishing the TME and modulating immune evasion mechanisms by altering the phenotype and effector activity of immune cells (27). Li et al. recently published a comprehensive review of the role of lactate in physiologic and pathological processes (20).

In hepatocellular carcinoma (HCC), transcriptomic data revealed a lactate metabolism-related gene signature (LMRGS) based on the expression of six genes (*FKTN, PDSS1, PET117, PS1, RARS1*, and *RNASEH1*). HCC cases with high LMRGS were associated with immune cell infiltration characterized by the presence of follicular helper T cells (Tfh), Tregs, and M0 macrophages. In contrast, cases with a low LMRGS were mainly infiltrated by resting NK cells, monocytes, and mast cells (28).

As mentioned earlier, T cells are the most potent mediators of the adaptive antitumor immune response and require high glucose consumption to effectively perform their effector functions. Glucose deprivation decreases the phosphorylation of p28 and JNK induced after TCR stimulation (29), leading to decreased production of IFN-γ, IL-2, and TNF-α, and Th1 differentiation on CD4+ T cells and impaired exocytosis of cytotoxic granules on cytotoxic T lymphocytes (CTLs). Some reports indicate that glucose deprivation allows the glycolytic enzyme GAPDH to bind the 3’UTR region of the IFN-γ transcript and promotes the loss of the open chromatin marks H3K9Ac and H3K27Ac in Th1-related genes, preventing their transcription (30, 31). Additionally, T cells express the lactate transporters SLC5A12 and SLC16A1, and exposure to this metabolite alters some functional activities such as glycolytic activity, motility, the cytotoxic activity of CTLs, and skew CD4+ T cells to Th17 phenotype, characteristic of persistent inflammatory responses (32, 33).

The hostile environmental features of the TME, such as the presence of anti-inflammatory cytokines and metabolic reprogramming, promote the accumulation of Tregs that favor tumor survival and progression. Tregs exert their suppressor activity on other immune cells through membrane-membrane interaction or by producing anti-inflammatory cytokines, such as IL-10 and TGF-β (34). In the glucose deprived, lactate enriched TME, Tregs are able to maintain their suppressor activity, since their metabolism is reprogrammed by the master transcription factor FOXP3 from glycolysis to OXPHOS (35). Interestingly, Watson et al. reported that Tregs display a heterogeneous metabolism of glucose and lactate, in which glucose-avid Tregs show lower suppressor activity than lactate-avid Tregs that promote immune suppression and tumor progression. Moreover, glucose starvation skews Tregs toward lactate consumption, and blockade of the lactate transporter MCT1 promotes the accumulation of glucose-avid Tregs that preserve the antitumor functions of CTLs (36).

These studies demonstrate that glucose depletion and lactate accumulation probably due to the increased metabolism of proliferating immune cells are sufficient to inhibit the effector functions of T cells and induce the accumulation of Tregs. It is tempting to speculate that this process is hijacked and taken one step forward by tumor and stromal cells as an immune evasion mechanism capable of inducing T cell dysfunction in the TME. Dysfunctional T cells can be classified in anergic, exhausted and senescent, and have been described to be induced by the hostile conditions of the TME and fail to eliminate tumor cells (37). As discussed in the next section, metabolic alterations are drivers of ICs expression, which are markers of T cell exhaustion. Furthermore, Tregs and tumor-derived γδ T cells have been reported to induce a senescent phenotype on effector T cells *in vitro* that differs from exhaustion and anergy. This effect is induced by glucose deprivation-mediated AMPK activation, and glucose supplementation or the blockade of glucose uptake in Tregs reverts the acquisition of the senescent phenotype by effector T cells (38).

Exhausted T cells are mainly induced by chronic antigen stimulation, show diminished effector function and high expression of ICs. Interestingly, it has been reported that T cells display characteristics of metabolic dysfunction even before the emergence of the exhausted phenotype. Thus, exhausted T cells show reduced glucose uptake, and transcriptional changes suggestive of glucose deprivation, as well as diminished mitochondrial function related to increased mitochondrial depolarization and production of ROS (39). As a response to TME variations, epigenetic modifications have been suggested as drivers of metabolic exhaustion. Transcription factors TOX and NR4A drive epigenetic changes characteristic of T cell exhaustion that may be promoted by conditions like hypoxia, glucose restriction, methionine deprivation, and ROS accumulation, and in turn promote the expression of some ICs (40, 41).

In comparison, cellular senescence is mainly related to telomere shortening due to repetitive division cycles. Although a senescence-like phenotype has been reported in multiple immune cells (42), it has been mostly studied on T cells that are characterized by loss of surface expression of costimulatory CD28 and telomere shortening due to their elevated proliferative rate (43, 44). Similar to other senescent cells, T cells upregulate their glycolytic metabolism in order to maintain the metabolic requirements of the senescent phenotype, like the senescent-associated secretory phenotype (SASP) (45). Interestingly, senescence in T cells strongly depends on their mitochondrial content. In particular, CD4+ T cells have been reported to better withstand cellular senescence due to higher mitochondrial content and oxidative metabolism, in comparison to CD8+ T cells (46). By contrast, senescent CD8+ T cells are able to acquire a NK cell-like phenotype that might be important for the removal of other senescent cells, and possibly tumor cells (47).

Although changes in the concentrations of glucose and lactate have been shown to inhibit the effector mechanisms and change the phenotype of T cells, more studies are needed to clarify if these metabolic alterations are capable of driving cell exhaustion or senescence in TILs.

Effective antitumor responses require the participation of several other immune cells that may also be impaired by the elevated glucose consumption in the TME. Similar to CD8+ T cells, NK cells require a highly glycolytic metabolism during activation. Increased glucose uptake and mTOR1-mediated glycolytic activity are required for their IFN-γ and granzyme B production. This is illustrated by the fact that some NK cells, especially the CD56^bright^ subpopulation, depend on glucose uptake through the GLUT1 transporter for IFN-γ production (48). Similarly, high lactate concentrations inhibit the activation of the transcription factor NFAT in both CD8+ T and NK cells, impairing their tumor infiltration and cytolytic effector functions. Brand et al. reported that, in a model of mice engrafted with low Lactate Dehydrogenase-expressing (LDH^low^) melanoma, tumor progression was reduced in an IFN-γ-dependent manner. LDH^low^ tumors showed higher NK and CD8+ T cell infiltration with increased IFN-γ and granzyme B expression, compared to the control group (49). Moreover, CD56^bright^ NK cells cultured in conditioned media from colorectal cancer metastasis undergo apoptosis induced by lactate-mediated intracellular pH decrease, mitochondrial stress, and accumulation of ROS (50). Based on these findings, we propose that, in addition to promoting immune evasion at the primary tumor, a high lactate production may facilitate the establishment of the premetastatic niche.

Two opposite phenotypes have been described in macrophages. The M1 phenotype shows antitumor activity that depends on glycolytic metabolism, with high oxygen consumption and pentose phosphate pathway (PPP), while the M2 phenotype mainly depends on OXPHOS (51). In this regard, glucose deprivation and lactate accumulation may skew macrophages toward the M2 phenotype, favoring immunosuppression. In fact, the M2 phenotype polarizing cytokine IL-4 inhibits mTOR signaling, which favors oxidative metabolism over glycolytic activity (52). Lactate has been reported to inhibit LPS-induced glycolytic activity on bone marrow-derived macrophages (BMDMs), resulting in lower expression of the cytokines IL-6, IL-12p40, and the co-stimulatory molecule CD40 (53), and to induce the expression of M2-specific markers, such as arginase-1, Mannose Receptor C-Type 1, CD206, and VEGF production, through ERK/STAT3 signaling (54). This effect seems to be dependent on the heterodimer of the odorant receptor Olfr78, and the G-protein-coupled receptor GRP132 since knockdown of Olfr78 prevents M2 polarization and promotes tumor infiltration by M1 macrophages and CD4+ and CD8+ T cells (55).

The effect of glucose concentration on DC maturation and the subsequent activation of T cell responses is less clear. On the one hand, maturation of DCs induced by TLR ligands or type I IFN favors a switch from oxidative to glycolytic metabolism. Reports indicate that glucose restriction limits the ability of DCs to produce IL-2 and express the co-stimulatory molecule CD86, which results in impaired CD4+ T cell activation. Moreover, the inhibitory effect of IL-10 has been reported to be due to the blockade of the metabolic shift toward glycolytic metabolism (56, 57). On the other hand, it has been reported that LPS-stimulated DCs under glucose deprivation express higher amounts of co-stimulatory molecules CD80 and CD86 and the cytokine IL-12. Consequently, glucose-restricted DCs exhibit higher capability of activating antigen-specific CD8+ T cells than DCs stimulated in normal glucose concentrations. The restriction of nutrients, such as glucose or amino acids, inhibits mTOR signaling, which prevents HIF-1α activation and the metabolic switch in DCs (58). These seemingly contradictory results may be explained by the dual function of DCs associated with their maturation stage. Immature DCs display high phagocytic activity for antigen capture in tissues, so these cells may require a high nutrient consumption to support this active process. After maturation and migration to the lymph nodes to present antigens to T cells, DCs may reduce their metabolism to avoid T cell inhibition due to nutrient depletion. This is supported by the fact that AKT activation and increased glycolysis are early events of DC activation (59) and that, after migrating to lymph nodes activated T cells deprive DCs of nutrients (58). In this sense, glucose depletion in the TME may impair DC maturation and migration to lymph nodes, preventing efficient T cell activation.

Regarding the effect of lactate on DCs, it has been reported that lactate produced by lung cancer cells inhibits IL-2 and type I IFN production and induces the expression of IL-10. As a result, DCs fail to correctly present antigens to induce efficient antitumor T cell responses (60). In particular, lactate impairs type I IFN production on plasmacytoid DCs (pDC) through GRP81 and MCTs (61). In the TME, granulocyte-macrophage colony stimulating factor (GM-CSF)-induced tolerogenic DCs inhibit glycolytic metabolism on T cells and promote the Treg phenotype through lactate production (62).

As in the case of tumor cells, MDSCs have been reported to exhibit significant metabolic plasticity. Based on dynamic metabolic flux analysis, it has been proposed that MDSCs are mainly dependent on glycolysis while maintaining high tricarboxylic acid (TCA) cycle with minimal PPP and OXPHOS activities (63). In *Staphylococcus aureus* infection, MDSCs have been shown to require an increased glucose uptake to undergo complete maturation. In contrast, a glucose-deficient environment, such as the TME, promotes the accumulation of immature and highly suppressive MDSCs. Reports indicate that MDSCs cultured in high glucose concentrations display a reduced ability to suppress CD4+ T cell proliferation (64). The dependence of PMN-MDSCs on glucose uptake relies on the expression of glucose transporter GLUT3; knockdown of this transporter reduces glucose uptake and promotes apoptosis, suggesting it as a possible therapeutic target to restore antitumor immunity (65).

Similarly, *in vitro* exposure of mouse bone marrow cells to IL-6, GM-CSF, and lactate enhances the induction of bone marrow-derived MDSCs that display high capacity to suppress T cell proliferation and cytotoxicity of NK cells (66). Radiotherapy has been reported to promote the Warburg effect inducing lactate secretion in a mouse model of PDAC. Tumor-derived lactate promotes MDSCs induction and activation through the GPR81/HIF-1α/STAT3 signaling pathway, suggesting the role of enhanced lactate secretion as a mechanism of radioresistance (67).

According to this information, progressive glucose deprivation and lactate accumulation in the TME due to increased glycolytic metabolism suppress the effector functions of immune cells and skew them toward anti-inflammatory/pro-resolving phenotypes, which supports the survival of tumor cells. The combination of treatments to inhibit the glycolytic activity of tumor and stromal cells to restore baseline glucose levels and the consequent decrease in lactate accumulation in the TME is a promising therapeutic strategy to reinvigorate the effector functions of tumor-infiltrating cells.

### Metabolism of amino acids

As discussed earlier, metabolic reprogramming in the TME is a hallmark of cancer and is not restricted to the Warburg effect. Rapid growth and multiplication of tumor cells require a high demand for other nutrients, such as amino acids. It has been reported that some cancer cells lack the ability to synthesize some amino acids, so they depend on the exogenous supply of these nutrients to maintain their development and metabolism (68). To meet this metabolic demand, cancer cells increase the expression of transporters and enzymes involved in amino acid synthesis and metabolism (69), which may promote the deprivation of these nutrients in the TME and thus, the impairment of the effector functions of antitumor immune cells.

#### Glutamine

Glucose-derived pyruvate is recognized as the main anaplerotic substrate for cell proliferation and homeostasis. However, when this molecule is limited, other substrates such as glutamine fulfill this requirement. Glutamine is the most abundant amino acid in the blood and is involved in multiple biosynthetic pathways, such as the production of precursors for synthesizing nucleic acids and maintaining the cellular redox state. Glutamate and ammonia are used as substrates by the enzyme glutamine synthetase (GS) for the intracellular synthesis of glutamine. However, glutamine is considered a conditionally essential amino acid for highly proliferative cells since internal synthesis is insufficient to fulfill the increased demand in immune and tumor cells. It has been reported that the loss or downregulation of GS turns ovarian cancer and oligodendroglioma cells dependent on exogenous glutamine to maintain their proliferation (70). To fulfill glutamine requirements, tumor cells overexpress major glutamine transporters, such as the alanine-serine-cysteine transporter 2 SLC1A5 (formerly known as ASCT2) and SLC38A2 (SNAT2) (71). In the case of T cells, it has been reported that in glucose deprivation, T cells increase glutaminolysis to maintain the anaplerotic production of TCA cycle intermediates and ATP,in an AMP-activated protein kinase (AMPK)-dependent manner (72).

As a result of the increased glutamine consumption by tumor and immune cells, glutamine availability is reduced in the TME, which acts as a suppressive signal that inhibits antitumor phenotypes while promotes pro-tumor phenotypes on immune cells. The competition between immune and tumor cells for glutamine is illustrated by the fact that T cells also require the expression of transporter ASCT2 for proper activation. Knockdown of this transporter dampens the activation of CD4+ T cells and their differentiation toward antitumor Th1 and Th17 phenotypes (73). In addition, it has been reported that glutamine deprivation impairs T cell proliferation and IL-2 and IFN-γ production, and this effect is dependent on kinase ERK (74). Likewise, NK cells require glutamine to maintain c-Myc activation, which is necessary to induce the metabolic switch and the production of IFN-γ and granzyme B (75). In fact, oral administration of glutamine has been proposed as a therapeutic strategy that enhances NK cell activity and reduces tumor growth in a fibrosarcoma rat model (76).

Regarding macrophages, glutaminolysis may be associated with the M2 phenotype. It has been reported that inhibition of glutamine metabolism in BMDMs through the inhibition of GS promotes M1 polarization by the accumulation of succinate and the activation of HIF-1α, resulting in an enhanced ability of macrophages to attract and activate T cells *in vitro* and *in vivo* (77). By contrast, it has been shown that α-ketoglutarate derived from glutaminolysis induces M2 gene expression through the activation of the histone demethylase JMJD3, promoting an open chromatin state at M2-specific gene promoters (78). Glutamine deprivation in the TME, induced by glutamine-addicted tumor cells, promotes IL-23 expression on Tumor-Associated Macrophages (TAMs) through HIF-1α activation, which is related to higher IL-10 and TGF-β production and recruitment of Tregs (79). MDSCs also display an increased glutamine uptake in the TME (80). In primary tumor and lung metastasis murine breast cancer models, Oh et al. demonstrated that glutamine antagonists reduce the number of MDSCs by inhibiting G-CSF expression, which promotes their differentiation to a pro-inflammatory phenotype. Moreover, inhibition of glutamine metabolism reduced the expression of IDO in tumor cells, TAMs, and MDSCs promoting T cell activation (81).

Therapeutic strategies that modulate glutamine metabolism on tumor cells and recover glutamine availability in the TME may act synergistically with other therapeutic options to inhibit tumor progression and reactivate the antitumor immune response.

#### Tryptophan and kynurenines

Tryptophan is another critical metabolite whose availability influences immune responses. About 95% of exogenous tryptophan is metabolized *via* the kynurenine pathway by the enzymes tryptophan-2,3-dioxygenase (TDO), regulating its plasmatic concentrations, and IDO1 acting in peripheral tissues. It has been proposed that IDO-mediated tryptophan metabolism regulates T cell responses to maintain immune privilege in the maternal-fetal interface (82). This process is co-opted by tumors cells to maintain an immunosuppressive environment since the overexpression of IDO has been reported in multiple tumors (83). Moreover, the accumulation of the IDO-derived metabolite l-kynurenine promotes the inhibition of immune cells *via* activation of the receptor AhR (84). In fact, AhR activation by microbiome-produced tryptophan catabolites promotes the suppressor functions of PDAC TAMs, which reduces tumor infiltration of CD8+ T cells (85).

IDO overexpression in tumors is associated to immune evasion and poor prognosis in cancer patients (86). Early studies indicated that IDO expression by tumor cells correlates with little or null T cell infiltration, and mice immunized with IDO1-expressing tumor cells fail to reject the tumor. Moreover, treatment with IDO inhibitors promotes tumor rejection (84, 87–89). IDO-mediated tryptophan catabolism is regarded as a powerful immunosuppressive mechanism. Culture in low tryptophan and high kynurenine concentrations inhibit the activity of T cells. T cells activated in these conditions display lower proliferation and are more prone to Fas-mediated apoptosis (90). CD8+ T cells co-cultured with IDO-expressing DCs show lower expression of the CD3ζ chain, decreased production of IL-2 and IFN-γ, and are devoid of cytotoxic activity. Regarding CD4+ T cells, reports indicate that cell cultures in low tryptophan and high kynurenine concentrations inhibit Th17 differentiation and promote Treg cell phenotype with increased expression of IL-10 and TGF-β (91, 92).

Overexpression of IDO has been reported in other cellular components of the TME to exacerbate tryptophan deficiency and accumulation of kynurenines. Both tumor cells and melanoma-associated fibroblasts (MAFs) produce COX-2 and IDO, inducing the expression of IL-10 in macrophages. Consequently, the pharmacological inhibition of COX-2 and IDO reverts the suppressive phenotype in macrophages (93). In addition, monocyte-derived macrophages induced by M-CSF upregulate IDO in response to IFN-γ or CD40L to deplete milieu tryptophan and suppress T cell responses to maintain peripheral tolerance (94). Although it has been suggested that monocyte-derived macrophages display a specialized tryptophan uptake system that may promote tryptophan deficiency in the TME (95), more studies are required to identify the molecules involved in this mechanism as possible therapeutic targets.

IDO expression has been widely reported in some subpopulations of DCs (96, 97). This expression is induced *via* interaction with Tregs, and IDO-expressing DCs can further promote the differentiation of Tregs, thus creating an amplifying immunoregulatory loop in the TME (96). In this regard, differentiation of Tregs from naïve CD4+CD25-T cells is promoted by IDO-derived kynurenine, and pharmacological inhibition of IDO prevents pDC-mediated induction of Tregs (98). Tryptophan deficiency in the TME may also impair the antitumor functions of DCs. Tryptophan-deprived DCs show decreased antigen uptake, decreased expression of the maturation markers CD40 and CD80, and increased expression of the inhibitory receptors ILT3 and ILT4. As a result, DCs show a reduced ability to activate T cells, thereby promoting Treg cell differentiation (99). Moreover, the tryptophan catabolite 3-hydroxyanthranilic acid reduces the phosphorylation of p38 and JNK, which prevents the maturation of mouse DCs, based on the expression of CD80, CD86, and CD40, and decreases the activation of T cells (100). IDO expression in human and mouse melanoma tumors has been associated with aggressive tumor growth that depends on the recruitment of highly suppressive CD11b+Gr1^int^ MDSCs mediated by Tregs (101). Moreover, GM-CSF derived from tumor cells has been reported to induce IDO expression on MDSCs (102) and IDO-expressing MDSCs promote the expansion of Tregs, that subsequently inhibit T cell proliferation and the antitumor immune response (103–105).

IL-2-activated NK cells in presence of l-kynurenine show impaired expression of the activating receptors NKp46 and NKG2D, lower production of IFN-γ and TNF-α, and reduced cytotoxicity against tumor cells (106). Furthermore, exposure to l-kynurenine promotes apoptosis in NK cells through ROS production (107). Controversially, kynurenine signaling through the AhR has been reported to promote the expression of the activating receptors NKp30, NKp46, perforin, and granzyme B in NK cells (108), which is supported by the observation that IDO1 inhibition impairs NK cell activity against tumor cells (109).

IDO inhibitors are currently under research, alone or in combination with ICIs. However, some clinical trials have shown that IDO inhibition does not improve the benefits of IC blockade alone. Several features have been proposed to impact the efficacy of inhibiting tryptophan catabolism, such as the participation of other enzymes, like TDO, the presence of multiple metabolites that drive AhR activation in the TME, and the augmented expression of IDO as a resistance mechanism to IC blockade (110).

#### Arginine

Arginine is synthesized from citrulline *via* the enzymes arginosuccinate synthase (ASS) and arginosuccinate lyase. Arginase is involved in the production of nitric oxide by the enzyme nitric oxide synthase (NOS), an agent involved in tumor development. Most solid tumors and leukemias lack the expression of the critical enzyme for arginine synthesis, due to methylation-induced transcriptional silencing, making them dependent on exogenous arginine. In addition, repression of the Argininosuccinate synthase 1 (ASS1) promoter by HIF-1α has also been implicated (111, 112). As a consequence, arginine deprivation induces cell arrest and tumor cell death (113). Thus, a high arginine uptake is mandatory for tumor cells, which may reduce its concentration in the TME and impair the antitumor activity of immune cells.

Activated T cells rapidly metabolize intracellular arginine to ornithine and citrulline, leading to enhanced CD4+ and CD8+ T cell survival and improved antitumor activity (114). In arginine deprivation, activated T cells fail to express cyclin D3 and cyclin-dependent kinase (cdk4), leading to arrest in the G0–G1 phase of the cell cycle (115). It has been shown that activated T cells that do not express ASS1 are unable to adapt to arginine deprivation, resulting in impaired metabolic processes due to reduced chromatin accessibility (116). In the case of NK cells, arginine deprivation impairs their proliferation, cytotoxic activity, and expression of the activating receptors NKp30 and NKp46 (117). Arginase derived from polymorphonuclear granulocytes depletes local arginine, resulting in decreased proliferation and IFN-γ secretion by NK cells (118), a process that might be supported by tumor cells.

Unlike T and NK cells, arginine deprivation does not affect the phagocytic activity, expression of activation markers, or cytokine production in macrophages (119). However, the dichotomy between the M1/M2 phenotypes of macrophages is mainly distinguished by their arginine metabolism. The antitumor M1 phenotype produces iNOS to convert arginine to nitric oxide (NO) and citrulline, while the M2 phenotype produces arginase to convert arginine to ornithine and urea and promotes immune evasion and tumor progression (120, 121).

Tumor-infiltrating MHC-II+/CD11b+/CD11c^high^ DCs (TIDCs) have been reported to suppress CD8+ T cell responses *via* the upregulation of arginase, inducing less proliferation and CD3ζ chain expression on T cells upon interaction (122). Moreover, arginine depletion in the TME promotes the accumulation of CD11b+Gr1+ MDSCs that suppress antitumor functions of T cells (123), and pharmacological inhibition of arg-1 suppresses the activity of G-MDSCs, restoring the production of IFN-γ and granzyme B by T cells and reducing tumor growth (124). Interestingly, arginine metabolites such as spermidine, produced by MDSCs, induce IDO1 expression on DCs, promoting a crosstalk between arginine and tryptophan metabolism that enhances immunosuppression in the TME (125). Like glutamine, oral arginine supplementation has been shown to reduce tumor growth in a breast cancer mouse model by decreasing the amount of tumor and splenic MDSCs. Moreover, arginine supplementation promoted the accumulation of activated macrophages, mature DCs, as well as activated CD4+ and CD8+ T cells, which resulted in the inhibition of tumor growth and enhanced survival of mice (126).

#### Adenosine

ATP is an ubiquitous intracellular molecule in cellular bioenergetics (127). Depending on the type of cell death, ATP is released from dying cells, and levels of extracellular ATP are proportional to the number of irreversibly damaged cells. Extracellular ATP is regarded as a DAMP that induces immune responses to eliminate the insult that causes cellular damage. To prevent exacerbated immune responses, extracellular ATP is eventually degraded by ectonucleotidases CD39 and CD73 to ADP and adenosine (128). Adenosine plays a dual role in cell biology, depending on its localization. Intracellular adenosine is involved in energy metabolism, nucleotide synthesis, and the methionine cycle, while extracellular adenosine acts as a signaling molecule related to immunosuppression. In the TME, conditions such as hypoxia, tissue disruption, inflammation, and overexpression of CD39 and CD73 promote adenosine accumulation, favoring the immunosuppressive state of the TME (129).

Hypoxia induces the expression of CD39 and CD73 in both tumor and stromal cells. In turn, adenosine signaling induces the expression of CD39 and CD73 in T cells, creating a positive feedback loop (130, 131). Interestingly, CD8+ T cells have been reported to support adenosine production by releasing EVs containing CD73, which degrade AMP and impair T cell proliferation upon activation (132). High adenosine concentrations inhibit different points of T cell activation. It has been reported that adenosine prevents the expression of high-affinity chain IL-2 receptor (CD25), the activation of kinase ZAP70, as well as synthesis of IL-2, TNF-α, and IFN-γ. In CTLs, adenosine signaling through A2AR results in decreased glycolytic activity and OXPHOS, which dampens essential functions such as adhesion to target cells and expression of effector molecules, such as Fas-L and perforin (133–135). Concerning NK cells, adenosine has been reported to inhibit their cytotoxic activity against tumor cells and their production of TNF-α, IFN-α, and GM-CSF (136). This inhibitory effect is reverted by blockade of CD39 and CD73 with antibodies, which restores the cytotoxicity of NK cell against tumor cell lines (137). Furthermore, depletion of the A2AR increases the percentages of mature NK cells with CD11b+CD27- phenotype in the TME, resulting in reduced tumor growth (138).

Adenosine not only promotes the M2 phenotype in macrophages by activating STAT3 and inducing arginase-1 and IL-10 expression (139), but also promotes the angiogenic role of M2 macrophages by promoting VEGF production (140, 141). Moreover, adenosine accumulation in the TME promotes the recruitment of TAM precursors that strengthen adenosine production by expressing CD39 and CD73 and impair the proliferation of T CD4+ cells (142). High concentrations of adenosine also alter the antitumor activity of DCs. DCs exposed to adenosine show lower production of TNF-α and CXCL10 and higher production of IL-10, inhibiting their ability to induce the Th1 phenotype in T cells (143). Similarly, it has been reported that the selective deletion of A2AR in the myeloid lineage delays tumor growth in mouse melanoma and lung cancer models. This effect is associated with higher expression of class II molecules and IL-12 in TAMs and lower production of IL-10 in macrophages, DCs, and MDSCs. As a result, NK cells and CD8+ T cells increase the production of IFN-γ and their cytotoxic activity against tumor cells (144). Furthermore, adenosine signaling through A2BR favors tumor growth by supporting the recruitment of CD11b+Gr1+ MDSCs that impairs tumor infiltration by CD8+ T and NKT cells and their production of TNF-α, IFN-γ, and granzyme B (145). Consequently, knockdown of CD73 diminishes GM-CSF in PDAC tumors, resulting in decreased circulating MDSCs and reduced tumor growth (146).

In summary, high nutrient consumption added to the accumulation of catabolites by tumor and stromal cells inhibit the activity of antitumor immune cells, while favors the recruitment and skewing to pro-tumoral phenotypes. The main alterations induced in tumor-infiltrating immune cells by metabolic alterations are summarized in **Table 1**. In early stages of tumor development, available nutrients support proliferation of the incipient tumor as well as the activation of an antitumor immune response. However, as tumor cells increase their nutrient consumption and secretion of by-products to maintain their uncontrolled proliferation, a metabolically hostile environment is gradually established. Tumor cells adapt and take advantage of this conditions to signal antitumor immune cells, such as M1 macrophages, NK, and T cells to shut down their effector mechanisms and turn into immunosuppressive/pro-resolving phenotypes, such as M2 macrophages and Tregs that favor tumor progression. Despite the enormous efforts to elucidate the effect of local metabolic alterations on immune cells, a deeper understanding is required to unravel the crosstalk between metabolic and immune cells for the development of more effective therapeutic strategies to increase the survival of cancer patients.

**Table 1:** Main alterations of tumor-infiltrating cells by metabolic alterations.

	Effect on immune cells				
Metabolic alterations in the TME	T cells	NK cells	Macrophages	Dendritic Cells	MDSCs
↓ Glucose	Impaired proliferation, cytotoxicity, and cytokine production (31) Tregs accumulation (36)	Impaired IFN-γ and granzyme B production (48)	Polarization towards M2 phenotype (51)	Impaired DC maturation, but strengthen T cell activation at lymph nodes (56–58)	Accumulation and survival in the TME (64)
↑ Lactate	Decreased motility, decreased glycolysis (33) Decreased cytotoxicity (29)	Decreased infiltration and IFN-γ and granzyme B production (49) ROS-mediated apoptosis (50)	Inhibition of glycolytic activity (53) Polarization towards M2 phenotype (54, 55)	Inhibition of IL-12 production and change to IL-10 (60) Inhibition of type I interferon production on pDCs (61)	Promotes IL-6 and GM-CSF induced MDSCs differentiation (66)
↓ Glutamine	Inhibition of Th1 and Th17 phenotypes (73) Impaired production of production of IL-12 and IFN-γ (74)	Impaired c-Myc activation Impaired IFN-γ and granzyme B production (75)	Polarization towards M2 phenotype (78) IL-23 production to promote Tregs (79)	–	Inhibition of IL-6 and GM-CSF-induced MDSCs differentiation Inhibition of IDO expression (81)
↓ Tryptophan/ ↑ Kynurenines	Impaired activation Sensitization to Fas-L induced apoptosis (90) Diminished expression of CD3ζ chain Diminished T CD8+ cytotoxicity (91) Skew towards Treg phenotype (92)	Impaired expression of activating receptors NKp46 and NKG2D, production of IFN-γ and TNF-α, and cytotoxicity against cancer cells (106) ROS-mediated apoptosis (107)	Promotes inhibitory functions of TAMs against CD8 + T cells (85) Induced IL-10 expression (93)	Impaired DC maturation. Impaired ability to activate T cells Enhanced ability to promote Treg phenotype (99, 100)	Recruitment of highly suppressive MDSCs (101)
↓ Arginine	Arrest in the cell cycle (115). Metabolic activity similar to quiescent cells (116)	Impaired mTOR activation Impaired production of IFN- γ (119) Impaired cytotoxicity and expression of the activating receptors NKp30 and NKp46 (117)	Unaffected phagocytic activity, secretion of cytokines and expression of activation markers (119) Arginine metabolism distinguishes between M1 and M2 phenotypes (120, 121)	Enhanced IDO expression (125). Upon interaction with T cells diminish their proliferation and expression of the CD3ζ chain (122)	Accumulation in the TME (126)
↑ Adenosine	Impaired TCR and CD28-mediated activation of ZAP-70 Impaired production of IL-2, interferon γ and TNF-α Impaired cytotoxicity Tregs recruitment (133–135)	Impaired cytotoxicity Impaired production of TNF-α, IFN-α, and GM-CSF (136)	Polarization towards M2 phenotype (139). Promotes VEGF secretion (140). Accumulation of CD39 and CD73-expressing TAMs (142)	Enhanced expression of IL-10. Impaired production of TNF-α and CXCL10 (143)	Recruitment to the TME (145, 146)

↑ High concentration; ↓ Low concentration or starvation.

The present review focuses on a better knowledge of the local alterations driven by the metabolism of tumor and tumor-associated stromal cells and how these local alterations disrupt the antitumor mechanisms of immune cells in the TME. However, processes such as aging and obesity that alter the systemic concentrations of nutrients are of key importance and must be addressed for the optimization of effective antitumor therapies.

## Metabolic reprogramming drives the expression of immune checkpoints

Cancer immunotherapy is a therapeutic strategy that aims to reactivate the patient’s antitumor immune response against tumor cells. These strategies include cancer vaccines, adoptive cell transfer, and immune checkpoint blockade (ICB). Immune checkpoints (ICs) are membrane proteins expressed mainly on immune cells that act as co-stimulatory or co-inhibitory receptors of the immune response, so that upon binding to their ligands, ICs can positively or negatively regulate the function of immune cells. As a mechanism of immune evasion, tumor cells commonly induce the overexpression of inhibitory ICs and their ligands in the TME (147, 148). Many ICs have been described and new information is continuously reported (149). In this study, we include information of the inhibitory ICs: CTLA-4, PD-1, LAG3, TIM3, VISTA, and TIGIT (**Table 2**) (150–171).

**Table 2:** Main ICs and their ligands expressed in the TME.

IC	Biological roles	Roles in cancer	Ligands	Ref.
CTLA-4 (CD152)	Negative regulator of naïve T cell activation Enhances suppressive activity of Tregs Competes with CD28 for its binding with CD80 and CD86 Mediates transendocytosis of its ligands	Induces expression of IDO Anti-CTLA-4 therapy depends on depletion of tumor-infiltrating Tregs	CD80 CD86	(150–154)
PD-1 (CD279)	Ubiquitously expressed on immune cells Highly stabilized by glycosylation Impairs activation, proliferation, and cytokine production of T and NK cells	Controls immune tolerance in the TME Main target of ICB	PD-L1 (CD274) PD-L2 (CD273)	(155–158)
LAG-3 (CD223)	Prevents TCR binding to MHC-II molecule Promotes Treg-mediated immunosuppression Key role in preventing autoimmune disorders	Promotes immune evasion of tumor cells Skews CD4+ T cells toward Treg phenotype Inhibits proliferation and maturation of DCs	MHC-II molecule Galectin-3 LSECtin	(159–161)
TIM-3 (HAVCR2)	Promotes Treg-mediated immune suppression Inhibits activity of Mφ and DCs	Marker of terminally exhausted T cells Favors M2 polarization on Mφ TIM-3 is upregulated in response to anti-PD-1 therapy	Galectin-9 (Gal-9) HMGB1 PtdSer CEACAM1	(162–165)
VISTA	Blocks early T cell activation Able to form homophilic interactions Mainly induced by hypoxia	Promotes induction of resting memory and exhausted phenotype on T cells VISTA is upregulated in response to ant-PD-1 therapy	VISTA VSIG3 PSGL-1 Gal-9	(166, 167)
TIGIT	Inhibits T cell activation Induces tolerogenic DCs Marker of terminally exhausted T and NK cells	Its expression is related to higher PD-1 expression on TILs Marker of specially suppressor Tregs Inhibits differentiation of CD4+ T cells toward Th1 and Th17 phenotype	PVR (CD155) PVRL2 (CD112) PVRL3 (CD113)	(168–171)

### Glucose/lactate

Multiple studies have demonstrated that glucose deprivation alone or combined with lactate exposure induces ICs expression on immune cells. Although the PD-1/PD-L1 axis is the most studied IC and the main therapeutic target of immunotherapies, alterations in the concentration of metabolites in the TME modulate the expression of many other ICs. Thus, combinatorial therapies that simultaneously target tumor glucose metabolism and the expression of ICs may restore the antitumor state of tumor-infiltrating immune cells.

As mentioned above, a scRNA-seq analysis of breast cancer patients has shown that tumor cells exhibit a gene signature associated with elevated glucose metabolism compared to normal epithelial cells. Interestingly, the highest glucose uptake in TME is displayed by exhausted CD8+ T cells, which displayed increased expression of the ICs PD-1, TIM-3, LAG3, and TIGIT (25). *In vitro*, culture in low glucose concentration induce the expression of PD-L1 on highly glycolytic PDAC cells and PD-1 on co-cultured CD8+ T cells. Knockdown of the enzyme phosphofructokinase-m (PFK-m) before tumor engraftment on mice reverted PD-1 expression on CD8+ TILs and the corresponding PD-L1 on tumor cells suggesting that this phenomenon was mediated by the high glycolytic activity of PDAC cells (26). Similarly, inhibition of glycolysis in renal cancer cell lines by the knockdown of HIF-1α, PFKFB3, or LDHA, or by culture under glucose deprivation induces PD-L1 expression, and glucose supplementation reverses this effect. Mechanistically, low glucose concentration promotes epithelial growth factor receptor (EGFR) expression, which induces PD-L1 expression *via* the EGFR/ERK1/2/c-Jun signaling pathway and PD-L1 stabilization through glycosylation (172, 173). Similarly, soluble mediators secreted by tumor cells, such as hyaluronan fragments, induce a metabolic shift toward aerobic glycolysis in tumor-infiltrating monocytes. This metabolic change causes the overexpression of the glycolytic enzyme PFKFB3, promoting the signaling by NF-κB, which increases PD-L1 expression. Inhibition of glycolysis by the glucose analog 2-deoxy-D-glucose (2-DG) reversed this effect (174).

Consequently, the pharmacological inhibition of Akt in esophageal cancer cell lines by ginsenoside Rh4 reduces the expression of crucial glycolytic enzymes, such as GLUT1, HK2, LDHA, PFKL, and PKM2, as well as the lactate production, which results in lower PD-L1 expression (175). Moreover, it has been reported that PKM2 is mandatory for PD-L1 expression in tumor, immune, and stromal cells in the TME and lymph nodes (176). Dimerization and nuclear translocation of PKM2, induced by M2 TAMs-derived TGF-β, allows its interaction with the nuclear factor STAT1 to promote the overexpression of PD-L1, event that impairs NK cell-mediated antitumor immune response (177). Concomitantly, lactate accumulation has been reported to induce PD-L1 expression in lung cancer cell lines in a dose-dependent manner. Lactate represses the activity of PKA, allowing the interaction of TAZ with the transcription factor TEAD, and their recruitment to the PD-L1 promoter (178). Similarly, in absence of lactate, activation of NF-κB prevents the transcription of the IC Galectin-9 (Gal-9) by binding to histone deacetylase HDAC3 in head and neck carcinoma cell lines. Thus, accumulation of PKM2-derived lactate induces the expression and secretion of Gal-9 (179).

Although the main inductor of the IC VISTA is the hypoxic microenvironment, lactate accumulation promotes VISTA-mediated immunosuppression through the acidification of the TME. VISTA suppresses T cell functions at physiologic pH, but this suppression is improved at acidic pH. The extracellular domain of VISTA is enriched with protonated histidine residues at acid pH, which allows its binding to P-selectin glycoprotein ligand-1 (PSGL-1), transmitting an inhibitory signal to T cells (180).

#### Glutamine

A RNA-seq analysis has demonstrated that tumor cells cultured in glutamine starvation express higher PD-L1 levels and secrete this IC in exosomes. Deprivation of glutamine inhibits the activity of Sarco/ER Ca2+-ATPase (SERCA), since it is an essential amino acid for glutathione synthesis. As a result, less Ca2+ is released from the endoplasmic reticulum, which promotes the activation of the NF-κB that induces PD-L1 expression. Therefore, inhibition of glutamine transport or GLS enzyme induces PD-L1 expression (181). In bladder and renal cancer cell lines, glutamine restriction induces EGFR activation and PD-L1 expression through the EGFR/ERK1/2/c-Jun signaling pathway (182, 183). Conversely, in natural killer T cell lymphoma cell lines, PD-L1 expression is reduced by overexpression of the glutamine transporter SLC1A1 (184).

In comparison, glutamine deprivation prevents PD-1 expression in T cells, since cell culture in this restricted conditions reduces PD-1 expression and promote IFN-γ production in CD8+ T cells (185). In fact, intravenous glutamine supplementation has been reported to reduce PD-1 expression on CD4+ and CD8+ T cells and PD-L1 expression on peripheral and splenic B cells and monocytes (186). In addition to the PD-1/PD-L1 axis, the IC B7/H3 is prone to be regulated by glutamine metabolism. Inhibition of glutamine uptake by an antagonist of the amino acid transporter ASCT2 (SLC1A5) promotes B7/H3 degradation through autophagy and ROS production in breast cancer cell lines, favoring the activation of tumor-infiltrating CTLs (187).

#### Tryptophan and kynurenines

As previously mentioned, overexpression of IDO in the TME promotes tryptophan depletion and the concomitant accumulation of kynurenines plays a vital role in the expression of ICs. Upon binding to kynurenines, AhR is translocated to the nucleus and binds to AHR-specific xenobiotic response elements (XREs). XREs are found in the promoters, of PDCD1 (PD-1), Lag3, Tim3, Klrg1, Ctla4, Btla, 2B4, CD160, and TIGIT, which promote the expression of these ICs (188). Thus, AhR activation by tobacco smoke induces PD-L1 expression in lung cancer cell lines and mouse models (189). In bladder cancer cells, IDO expression correlates with epithelial–mesenchymal transition (EMT) and PD-L1 overexpression *via* the IL-6/STAT3 signaling pathway (190).

T cells increase tryptophan metabolism upon activation, and its deprivation has been reported to induce PD-1 expression in Jurkat cells and mouse T cells. The absence of tryptophan prevents ubiquitylation and the subsequent degradation of the transcription factor NFAT-1, which promotes PD-1 expression. Consequently, the restoration of tryptophan concentrations or inhibition of IDO block PD-1 expression (191). Moreover, kynurenine produced by tumor cells induces PD-1 expression in human and mouse CD8+ T cells through nuclear translocation of the receptor AhR (192). Results from the assay for transposase-accessible chromatin sequencing (ATAC-seq) in human ovarian cancer cells revealed that exposure to kynurenine promotes chromatin accessibility of PD-1 regulatory regions, allowing AhR to induce its transcription (188). As discussed earlier, low tryptophan and high kynurenine conditions promote the differentiation of T cells toward a CD4+CD25+Foxp3+ regulatory phenotype. Additionally, Tregs exposed to low tryptophan and high kynurenine concentrations upregulate CTLA-4 and BTLA (91). CTLA-4 overexpression has been reported in CD4+CD25+ T cells co-cultured with IDO-expressing acute myeloid leukemia (AML) cells, and this effect is completely abrogated by the IDO-inhibitor 1-methyl tryptophan (1-MT) (193).

The report of Wu and Zhu illustrates the effect of IDO expression and tryptophan metabolism on the inhibition of antitumor functions of T cells and ICs expression*. In vitro*, exposure to kynurenine inhibits IFN-γ and TNF-α production in CD8+ T cells; *in vivo*, IDO knockdown in colon carcinoma cell lines before engraftment reduces the expression of the ICs: PD-1, CTLA-4, and LAG3 in CD8+ TILs. Moreover, in colorectal cancer patients, the expression of these ICs on CD8+ T cells positively correlates with serum kynurenine concentrations (194). In addition to PD-1, culture of CD8+ T cells in kynurenine-enriched media derived from IDO-expressing mouse cells induces the expression of the inhibitory receptors KLRG1 and TIM-3 (188), so that targeting the IDO pathway may prevent the expression of multiple ICs.

The expression and secretion of the recently incorporated IC HLA-G is also regulated by the availability of tryptophan and its catabolites. Macrophages and DCs, derived from healthy donor monocytes, and matured in the presence of tryptophan or its catabolites express high levels of HLA-G. Kynurenine is the metabolite that induces the highest surface expression of HLA-G in DCs, whereas 3-hydroxy anthranilic acid induces this same effect in macrophages. Furthermore, kynurenine induces the shedding of HLA-G by immature and mature DCs to impair T cell proliferation (195, 196).

The effect of tryptophan and its IDO-derived catabolites on IC expression opens the possibility for combinatorial therapies that target this pathway to improve the efficacy of immunotherapy or conventional therapies. In this regard, in a mouse model of Lewis lung cancer, IDO inhibition by oral administration of 1-MT synergizes with radiotherapy by reducing the expression of PD-1/PD-L1, TIM-3, BTLA, and Gal-9 to restore antitumor immune response and inhibit tumor progression (197). With respect to VISTA, knockdown or pharmacological inhibition of the AhR reduces VISTA expression in melanoma cell lines. Interestingly, metformin treatment inhibits AhR signaling and VISTA expression *in vitro* and in melanoma mouse models (198).

#### Arginine

As was indicated earlier, metabolic alterations and the induction of IC expression in immune cells are not only mediated by tumor cells. Simultaneous expression of VEGF, IL-10, and arg-1 on tumor cells induces TIM-3 expression on BMDCs and tumor-associated DCs. The inhibition of these molecules diminishes the induction of TIM-3 on DCs by tumor cell-conditioned media (199). Cancer-associated fibroblasts (CAFs) have been reported to inhibit antitumor immune responses. CAFs inhibit T cell activation and CD8+ T cell proliferation by expressing high levels of arg-1 enzyme, and the ICs VISTA and HVEM. The inhibitory effects of MAFs may be mediated by arginine depletion. MAFs-conditioned media induces the expression of TIGIT and BTLA on CD8+ T cells, and the inhibition of arginine metabolism by knockdown or pharmacological inhibition of arg-1 reverts this effect (200).

### Adenosine

In addition to the direct suppression of the antitumor functions of immune cells by adenosine accumulation, especially due to the overexpression of CD39 and CD73, IC expression is enhanced by adenosine signaling. In biopsies from PDAC patients, CD73 expression on tumor and stromal cells correlates with PD-L1 expression on tumor cells (201). Additionally, in biopsies from NSCLC patients, CD39+ CAFs positively correlate with PD-L1 expression on tumor cells (202). Similarly, the activation of the A2AR induces PD-1 expression, but not CTLA-4, on CD8+ and CD4+ FOXP3+ TILs *in vitro*, and in a mouse model of colon adenocarcinoma. Consequently, CD8+ TILs from CD73 deficient mice show lower PD-1 expression compared to T cells from wild-type mice (203), and deletion of the A2AR results in lower PD-1 expression in CD8+ TILs in a melanoma mouse model. Nevertheless, the deletion of A2AR in T cells reduces their survival, resulting in enhanced tumor growth (204).

By using mixed lymphocyte reactions with T cells and DCs derived from mouse, it has been shown that A2AR activation by an agonist reduces T cell proliferation, while inducing PD-1 and CTLA-4 expression (205). Moreover, A2AR stimulation induces the expansion of Tregs in the TME, which further increases adenosine accumulation, since A2AR agonist-induced Tregs to express CD39 and CD73. These A2AR-induced Tregs show high expression of CTLA-4, which plays a crucial role in their inhibitory activity (206). Contrary to the effect of adenosine on ICs expression, ATP binds to its P2X7R on monocytes to inhibit the shedding of soluble HLA-G (207). In the context of the TME, adenosine accumulation due to overexpression of CD39 and CD73 ectonucleotidases may inhibit the stimulatory effect of extracellular ATP, promoting an increased production of both membrane and soluble HLA-G to limit antitumor immune responses.

Regarding combinatorial therapies that target adenosine signaling to improve the response to immunotherapies, oral administration of A2AR antagonists reduces PD-1 and LAG-3 expression on activated CD8+CD44+ T cells within tumor-draining lymph nodes (dLNs) and Tregs infiltrating tumors in a mouse melanoma model (208).

## Immune checkpoints feedback the metabolic alterations in the TME

### PD-1/PD-L1

Metabolically, PD-1 signaling inhibits T cell activation by altering metabolic reprogramming induced by TCR-mediated antigen recognition. It has been demonstrated that, when activation occurs in presence of PD-1 interacting with recombinant PD-L1, T cells show diminished glucose uptake and glycolytic capability due to a reduction in the expression of Glut1 transporter and HK2. Furthermore, PD-1 inhibits T cell capability of uptaking and utilizing amino acids such as valine and glutamine while promoting FAO by enhancing the expression of the carnitine pamitoyltransferase (CPT1A) and desnutrin/adipose triglyceride lipase (ATGL) (209). Exposition of PD-1 positive esophageal adenocarcinoma cell lines to the anti-PD-1 pembrolizumab augments glycolytic reserve by upregulating the expression of GLUT-1.

Similarly, PD-1 blockade increases GLUT1 expression on CD8+ T cells from B cell lymphoblastic leukemia-bearing animals. However, PD-1 blockade was insufficient to restore the antitumor functions of T cells, suggesting the existence of additional immunosuppressive or compensatory mechanisms that impair antitumor immunity (210). In this regard, it has been reported that resistance to anti-PD-1 treatment is due to increased lactic acid in TME, which promotes PD-1 expression of Tregs in intrahepatic tumors. Furthermore, resistance to anti-PD-1 treatment can be overcome by hindering lactate metabolism through inhibition of LDHA or MCT1 on Tregs (211). Moreover, knockdown of the glycolytic enzyme PKM2 in PDAC cells promotes NK cell infiltration, production of IFN-γ, granzyme B, and NKp46, and response to anti-PD-1 treatment (177).

These results show that metabolic modulations in TME improve the efficacy of ICIs, suggesting that IC blockade induces metabolic changes that impact the use of combination therapy. In addition, it has been suggested that PD-1 engagement impairs metabolic functions beyond glycolytic activity. RNA-seq and Gene Ontology analysis revealed that PD-1 signaling triggers a specific transcriptional program in CD8+ T cells involved in altered amino acid, nucleotide, and carbohydrate metabolism, as well as altered TCA cycle and OXPHOS. Furthermore, PD-1 affects the expression of genes involved in the structure and function of mitochondria, resulting in reduced mitochondria number and cristae length (212). Metabolic alterations driving T cell exhaustion after PD-1 engagement may be due to PD-1-mediated inhibition of the peroxisome proliferator-activated receptor-gamma co-activator (PGC)-1α, acting as a regulator of genes involved in energy metabolism and mitochondrial biogenesis (213). Moreover, PD-1 inhibitory signaling shifts metabolism away from aerobic glycolysis and glutaminolysis and forces T cell to events of anaplerotic input to the TCA cycle, mainly at acetyl-CoA and succinyl-CoA, also preventing the *de novo* nucleoside phosphate synthesis accompanied by decreased mTORC1 signaling (214).

Aside from T cells, PD-1 signaling causes metabolic dysfunctions in monocytes and macrophages. Monocytes isolated from Chronic Lymphocytic Leukemia (CLL) show diminished glucose uptake and lactate production. As discussed above, M1 Macrophages rely on aerobic glycolysis for their antitumoral functions. CLL monocytes show enhanced PD-1 expression compared to healthy donors, and ligation with recombinant PD-L1 diminishes monocytes switch to aerobic glycolysis, while anti-PD-L1 blockade reverts this effect, promoting phagocytosis of tumor cells (215). Interestingly, it is known that PD-1 expression on TAM of the TME can be induced by CAFs (216). In addition to PD-1-mediated metabolic alterations described in T cells and monocytes, in a melanoma mouse model it has been shown that PD-1 knockout reduces the accumulation of granulocyte and monocyte precursors, as well as the immunosuppressive activity of MDSCs, in part through metabolic alterations. In PD-1 knockout tumor-bearing mice, glucose uptake and mitochondrial biogenesis were elevated in myeloid progenitors, and it was suggested that, in PD-1 deficiency, glycolytic activity is progressively switched to mitochondrial metabolism. PD-1-deficient myeloid progenitors display increased metabolic intermediates of glycolysis, PPP, TCA cycle, and elevated cholesterol, resulting in enhanced differentiation toward effector monocytic/macrophage and DCs, thus promoting antitumor responses (217). Whereas PD-1/PD-L1 axis-mediated immune suppression is commonly attributed to PD-1 signaling, PD-L1 is known to mediate intracellular signaling that promotes cancer progression, immune escape, and metabolic reprogramming (218).

In patients with NSCLC, 2-Deoxy-2-[fluorine-18] fluoro-D-glucose (2-FDG) uptake has been reported to be higher in tumors with high PD-L1 expression, suggesting an interplay between PD-L1 expression and glucose uptake. Moreover, it has been suggested that an elevated glycolytic metabolism might be used as a prognostic biomarker for ICI treatment (219). In cervical cancer cell lines, it has been reported that PD-L1 promotes glucose metabolism and lactate secretion by interacting with integrin β4 (ITGB4), and suppressing SIRT3, resulting in the augmented expression of glycolytic enzymes HK2 and LDHA and of transporters GLUT1 and GLUT4 (220). Similar results have been reported in AML cell lines, where genes and the corresponding proteins associated with glucose metabolisms, such as ALDOA, PGK1, LDHA, and HK2, are highly expressed when PD-L1 is overexpressed (221). In a mouse model of sarcoma, it has been shown that monoclonal antibody blockade of CTLA-4, PD-1, or PD-L1 augments glucose availability in the TME and glycolytic metabolism in T cells by restoring mTOR-mediated signaling, as well as the activity of the enzyme glutamate dehydrogenase (Glud1). PD-L1 knockdown and antibody blockade diminish glucose metabolism and Akt/mTOR signaling in tumor cells without affecting proliferation *in vitro* or tumor growth in RAG^-/-^ mice. These results suggest that ICIs might revert metabolic alterations in tumor cells, allowing an enhanced nutrient availability in the TME to restore the functionality of antitumor immune cells (222). Mechanistically, PD-L1 might promote glycolysis by enhancing the expression of the glycolytic enzyme PFKFB3 since PD-L1 knockdown by a small interfering RNA also diminishes the expression of this enzyme (172).

As discussed earlier, lactate accumulation in the TME also impairs immune cell function and response to immunotherapy. For instance, lung cancer cells A549 exposed to high lactate concentration inhibit IFN-γ production and induce apoptosis of co-cultured Jurkat T cells. Interestingly, treatment with PD-L1-blocking antibody reverses the indicated effects (178), suggesting that anti-PD-L1 treatment modulates the inhibitory effect of tumor-derived lactate on immune cells. In addition, the inhibition of glutamine metabolism by a competitive antagonist of transmembrane glutamine flux or a GLS inhibitor, in combination with anti-PD-1 or anti-PD-L1 antibodies, has been shown to reduce tumor growth in breast and colon cancer mouse models to a greater extent than monotherapies (181, 187).

Interestingly, IDO-mediated tryptophan metabolism in the TME has been proposed as a major mechanism for resistance to ICIs. High IDO expression in macrophages and endothelial cells in TME are related to anti-PD-1 non-responsiveness in metastatic renal cell carcinoma and sarcomas patients undergoing anti-PD-1 treatment with pembrolizumab or nivolumab antibodies (223, 224). As mentioned earlier, the accumulation of IDO-derived kynurenine in the TME is associated with the upregulation of multiple immune checkpoints that may contribute to anti-PD-1 resistance (188). Combined checkpoint blockade therapy is becoming increasingly important, especially regarding cancers in advanced stages or associated with poor prognosis. For instance, a recent study in HER2-overexpressing cancers showed that using a bispecific antibody, simultaneously targeting PD-1 and HER2, represents a new promising approach for treating late-stage metastatic HER2-positive cancers (225).

### CTLA-4

One of the first relationships reported between ICs and metabolism was the influence of CTLA-4 on IDO expression (226, 227). *In vitro* stimulation of DCs with recombinant CTLA-4 showed an increased tryptophan metabolism to kynurenine, and IDO inhibitor 1-MT reversed this effect. Moreover, CTLA-4 stimulation induces IDO expression in DCs similarly to IFN-γ (228). Consequently, mice with a Treg-specific CTLA-4 knockdown show reduced IDO expression in CD11c+ mesenteric DC and reduced kynurenine production by spleen-isolated DCs (229). In the context of the TME, CTLA-4 expressing Tregs induced by metabolic alterations, may promote IDO expression in tumor-infiltrating DCs, reinforcing immune suppression. In T cells, CTLA-4 signaling inhibits glycolysis without inducing FAO; in contrast to PD-1 signaling, the authors suggest that CTLA-4 does not induce metabolic alterations related to T cell exhaustion but preserves the metabolic profile of unstimulated T cells (209). In addition, IDO activity in the peripheral blood of melanoma patients increases PD-L1 expression in circulating CTLs. This IDO activity is associated with a CTLA-4 increase in Tregs. This IDO/PD-L1/CTLA-4 interplay is associated with a negative prognosis of cancer patients, showing that the expression of IDO, PD-L1, and CTLA-4 is strongly interconnected (230).

Regarding ICIs, ipilimumab-mediated CTLA-4 blockade promotes immune cells metabolic fitness and infiltration. Moreover, ipilimumab treatment induces the functional destabilization of tumor-infiltrating Tregs by impairing cell glycolysis and CD28 signaling (231). It has been reported that anti-CTLA-4 monotherapy produces durable responses in many cancers, mainly in melanoma (232). However, combining anti-CTLA-4 antibodies with other therapeutic strategies, such as chemotherapy or radiotherapy, increases their efficacy (233–236). In fact, the modulation of TME by propranolol increases tumor T cell infiltration and the efficacy of anti-CTLA-4 treatment (237).

Research is still in progress to enhance anti-CTLA-4 therapy response. Notably, the modulation of the metabolic conditions in the TME may enhance response to ICIs in cancer patients. For instance, PD-1 or CTLA-4 inhibition has been reported to synergize with the blockade of adenosine metabolism to inhibit tumor growth in colon cancer and sarcoma mouse models. Targeting adenosine production by CD73 blockade combined with PD-1 or CTLA-4 inhibition promotes tumor regression in a manner dependent on IFN-γ and CD8+ T cells (203).

### LAG-3

LAG-3 expression has been reported to impair the metabolic shift of mouse T cells toward glycolysis, and its deficiency has been shown to augment metabolic fitness by promoting oxygen consumption and glycolytic activity. Conversely, LAG-3 expression in CD4+ T cells impairs mitochondrial biogenesis by altering the AMPK/Sirt-1 pathway, resulting in a diminished proliferation of CD4+ T cells and high IL-7 dependence (238). Similarly, LAG-3 deletion in mouse BMDCs has been reported to increase their glycolytic activity and FAO, which can be counteracted by IL-10. This enhanced metabolic switch in LAG3^-/-^BMDCs increases their ability to induce Th1-like responses, promoting antitumor immunity (239).

### TIM-3/Gal-9

In Jurkat T cells, TIM-3 overexpression has been reported to diminish glucose consumption and lactate production. TIM-3 expression downregulates Glut-1, while TIM-3 knockdown has the opposite effect. Interestingly, TIM-3 did not affect glutamine consumption, glutamate release, mitochondrial mass, ROS production, or membrane potential (240). Conversely, in Tregs, TIM-3 induces a metabolic shift from OXPHOS toward glycolysis while decreasing mitochondrial mass and membrane potential. TIM-3 expression in Tregs promotes tumor progression and exhaustion of CD8+ T cells enhancing their suppressive activity and IL-10 production (241).

Regarding myeloid cells, Gal-9 or antibody-mediated TIM-3 stimulation in THP-1 cells has been reported to induce mTOR phosphorylation, HIF-1α expression, as well as enhanced glycolytic activity and VEGF secretion by activating the PLC-1/PI3K/mTOR signaling pathway (242). Conversely, stimulation with a TIM-3 agonist in a mouse macrophage cell line has been reported to inhibit glucose uptake and lactate production by inhibiting HK2 expression, resulting in diminished production of TNF-α and IL-1β (243). As mentioned earlier, macrophages depend on glycolysis to differentiate into the antitumoral M1 phenotype, and TIM-3 expression may prevent M1 differentiation and promote the pro-tumoral M2 phenotype.

### TIGIT

In CD8+ T cells, the downregulation of GLUT1, HK1, HK2, GAPDH, PKM2, and HIF-1α metabolism-associated genes correlates with the expression of TIGIT. In addition, CD8+ T cells expressing TIGIT show reduced activation of the Akt/mTOR signaling pathway. Co-culture of gastric cancer cells with CD8+ T cells induces TIGIT expression and metabolic impairment, while the blockade of the TIGIT/CD155 axis restores normal metabolic functions in T cells and promotes antitumor immune response (244). Similarly, T cell dysfunction in colorectal cancer has been related to diminished glucose metabolism since TIGIT expression, and metabolic alterations induced by colorectal cancer cells are restored by antibody-mediated TIGIT blockade (245). Anti-TIGIT monotherapy has shown encouraging results in the treatment of diverse cancers, and combining TIGIT blockade with the inhibition of adenosine production, restores NK cell-mediated AML cell killing, which might enhance treatment efficacy (246).

## Targeting the crosstalk between metabolic reprogramming and ICs

Since the middle of the last century, metabolic inhibitors have represented a promising therapeutic alternative for treating several cancers, including brain, lung, breast, skin, and hematological cancers. However, the administration of metabolic inhibitors as monotherapy is insufficient since most tumors do not rely on a single metabolic pathway to meet their energetic demands (247). Additionally, the administration of these compounds represents a challenge due to off-target effects and toxicity in non-tumoral cells, narrowing the therapeutic index. Despite these hurdles, it seems promising that administering metabolic inhibitors in combination with chemotherapy, targeted therapy, or immunotherapy could circumvent the challenges of treatment failure. In this setting, as we discussed previously, the feedback established between metabolic reprogramming and immune checkpoint molecules represents a potential target to treat cancer and enhance the outcome of patients. In the present section, we discuss the current knowledge regarding the combination of both treatment options.

As mentioned above, tumor and stromal cells increase the expression of the enzyme IDO1, which metabolizes tryptophan into the immunosuppressive kynurenine. Several studies report that increased levels of IDO1 expression correlate with altered function of immune cells or accumulation of cells with immunosuppressive activity, which is associated with poor survival (248–251). Due to these therapeutic implications, several IDO inhibitors have been developed, showing promising results in pre-clinical models. For example, studies have reported that small-molecule IDO inhibitors synergize with immunotherapy based on the administration of monoclonal antibodies against ICs. To support this notion, administration of epacadostat in co-cultures conformed of human allogenic lymphocytes with DCs and tumor cells showed an increase in the number and activity of T and NK cells and a reduction in the proportion of immunoregulatory cells (252). Recent evidence from clinical trials demonstrates that epacadostat in combination with ICIs against PD-1 resulted in a well-tolerated combination regimen and improved progression-free survival/overall survival (253). In addition, a phase II trial reported that a combination of IDO inhibitor indoximod plus anti-PD1/PDL-1 antibodies pembrolizumab, nivolumab, and ipilimumab resulted in increased progression-free survival and a better response in patients with advanced melanoma (254). Despite these promising results, further clinical trials should be undertaken to conclude the efficacy of the combination therapy using IDO and ICIs.

However, IDO expression is not only responsible for consuming essential amino acids, such as tryptophan. As mentioned in previous sections, the tumor cell catabolism of glutamine and arginine starve tumor-infiltrating immune cells, causing a disruption in their activation and promoting their demise or differentiation into immunosuppressive subsets. For example, recent evidence from Varghese et al. demonstrates that the inhibition of glutaminase with telaglenastat improved the tumor-killing capacity of autologous patient-derived T cells against melanoma. Interestingly, authors reported that, in mice, combination of telaglenastat with immune checkpoint inhibitors against PD-1 or CTLA-4 increased the number of tumor-infiltrating T cells and the expression of genes associated with IFN-γ signaling (255). In the case of arginine, a recent study employing co-cultures of the Lewis cell line reported that administration of OAT-1746, an arginase inhibitor, restores CD4+ and CD8+ T cell activation by increasing CD3ζ chain expression. In addition*, in vivo* assays showed that the combination of OAT-1746 with anti-PD-1 antibody slightly increased the survival of mice compared to groups treated with the immune checkpoint inhibitor alone (256). In support of these findings, Pilanc et al. reported that, in a mouse model of glioma, this novel small-molecule arginase inhibitor in combination with immunotherapy also reduced tumor growth (257). Currently, no clinical trials evaluate the efficacy and safety of combining glutaminase or arginase inhibitors with immune checkpoint inhibitors in human cancer patients. These findings highlight the need for further clinical trials to evaluate the effect of combining glutaminase or arginase inhibitors with ICIs and to test tolerability and response in cancer patients.

In addition, to deplete and consume essential amino acids, tumor cells also catabolize glucose *via* aerobic glycolysis to produce lactate by the action of the LDH enzyme. To avoid the accumulation and acidification of the cytoplasm, tumor cells overexpress MCT transporters, specifically MCT1, on their surface to promote lactate extrusion into the TME. As was mentioned above, lactate accumulation in the TME favors the outgrowth of tumor cells and, more importantly, acts as an immunosuppressive metabolite disrupting the activity of immune effector cells and favoring the recruitment of MDSCs (258). For this reason, the inhibition of lactate transport into the TME represents an attractive strategy to restore the antitumor immune response. In a recent study, Babari et al. reported in a mouse xenograft of Raji cells that the administration of the MCT1 inhibitor AZD3965 inhibited tumor growth and promoted its infiltration by NK and monocyte-derived DCs cells. Interestingly, these tumor-infiltrating immune cells displayed high expression of PD-L1, suggesting the induction of an immunoregulatory phenotype (259). These findings suggest that combining MCT1 inhibitor AZD3965 with monoclonal antibodies against PD-L1 might be a promising therapeutic alternative to decrease tumor growth and restore the antitumor immune response. Currently, one orally bioavailable MCT1 inhibitor is under study in a phase I clinical trial showing promising results (260). For this, further clinical trials are required to test our proposal.

Another attractive point of metabolic inhibition is the enzyme IDH, which catalyzes the conversion of isocitrate into α-ketoglutarate depending on NADP. Reports indicate that isoforms (IDH1 and IDH2) of this enzyme are often mutated in some cancers, gaining a new function that results in the production of the immunosuppressive D-2-hydroxyglutarate (D2HG) (261). Currently, two IDH inhibitors approved by the FDA for treating AML and second-generation inhibitors are under clinical trials for treating gliomas (247). In the last case, a recent study reported in a mouse glioma that administration of AGI-5198, an IDH inhibitor, induced immunogenic cell death accompanied by increased expression of PD-L1 (262).

### Metabolic alterations at systemic level

Once we have analyzed current immunometabolism studies that comprehend the local impact of metabolic reprogramming on the antitumor immune response, it is important to highlight that systemic alterations of nutrients availability may also modify the functions of immune cells and impact the effectiveness of immunotherapies. Aging and obesity are two processes in which the systemic availability of nutrients is modified, and the normal functions of immune cells may be altered in consequence. For instance, aging has been related to changes in blood concentrations of amino acids and lipids (263), while obesity is related to metabolic alterations such as hyperglycemia, dyslipidemia, and insulin resistance (264). In fact, both aging and obesity have been reported to involve a low-grade chronic inflammation that alters the anti-tumor innate and adaptive immune responses (265). Excellent reviews have addressed the metabolic impact of these conditions on immune cells (266, 267).

Aging is a physiological process in which the biological functions of an individual slowly deteriorate with age. Immunosenescence is the term that describes the alterations on the immune system related to aging that impair its ability to respond against pathogens and cancer cells. Alterations on the metabolism and effector functions of multiple immune cells that may alter the antitumor response have been related to aging (266). For instance, a predisposition toward myelopoiesis has been suggested in aged mice, while lymphopoiesis is reduced (268). Regarding the metabolic changes related to immunosenescence, an increased glycolytic metabolism has been reported on monocytes, macrophages, and T cells from elderly mice. This effect is mediated by ROS accumulation that drives HIF-1α and NF-κB activation (269, 270). In addition, in adipose tissue macrophages from elderly mice it has been shown a decrease in FAO, driven by decreased PPARγ expression, which promotes an increased secretion of proinflammatory factors (271).

Systemic changes in the availability and metabolism of amino acids have also been related with aging, which may impair the antitumor immune responses as discussed above. On the one hand, the concentration of glutamine is reduced with ageing, while accumulation of ROS impairs the activity of glutamine synthetase (272).On the other hand, ageing increases IDO activity, which is reflected in increased kynurenine and reduced tryptophan concentrations in elderly (273). Additionally, the expression of the asparagine transporter SLC7A2 has been reported to decrease in macrophages from elderly mice (274).

Similarly, obesity-induced inflammation has been regarded as an important risk factor for cancer development, and secondary conditions such as insulin resistance, hyperglycemia and dyslipidemia have been related to tumor growth (264). Paradoxically, obesity has been related to enhanced response to ICB in tumor-bearing mice and cancer patients (275).

At the metabolic level, the two main adipokines altered in obesity display opposing roles in the activation of T cells. On the one hand, leptin is necessary for normal T cell proliferation, glycolytic metabolism, and production of IFN-γ, and IL-2 (276), and it has been reported to promote M1 polarization on macrophages, reduce MDSCs, and increase the effectiveness of ICB (277). On the other hand, adiponectin has been reported to impair the glycolytic metabolism of Th1 and Th17 cells (278). In addition, a mouse model revealed that diet-induced dyslipidemia impairs mTOR1 signaling in Tregs, which results in reduced glycolytic metabolism and increased FAO (279). Moreover, high fat diet consumption has been related to induction of an exhausted phenotype on T cells from the white adipose tissue, and overexpression of the IC BTLA (280).

At the TME, high fat diet has been reported to alter the expression of activation markers on T cell, probably related to a reduction on GLUT1 expression, while promoting fatty acid metabolism on tumor cells (281). This effect may be due to adipocyte derived leptin that impairs glycolytic metabolism and promotes FAO on TILs through activation of STAT3. In fact, tumor-infiltrating CD8+ T cells from obese breast cancer patients display lower expression of granzyme B compared to T cells from lean patients (282).

## Concluding remarks and perspectives

In incipient tumors, immune cells are recruited to an environment rich in nutrients and inflammatory stimulus, such as tumor antigens, DAMPs, and inflammatory cytokines. This environment strongly resembles immune responses against external pathogens. In the course of removing an insult, immune cells consume available nutrients and produce a spectrum of by-products that gradually change the surrounding milieu. As a result, the removal of the inflammatory stimulus, together with reduced nutrient availability and accumulation of by-products, signal immune cells to interrupt their pro-inflammatory programs and shift their phenotype toward anti-inflammatory programs, initiating the resolution phase of inflammation.

**Figure 1:**
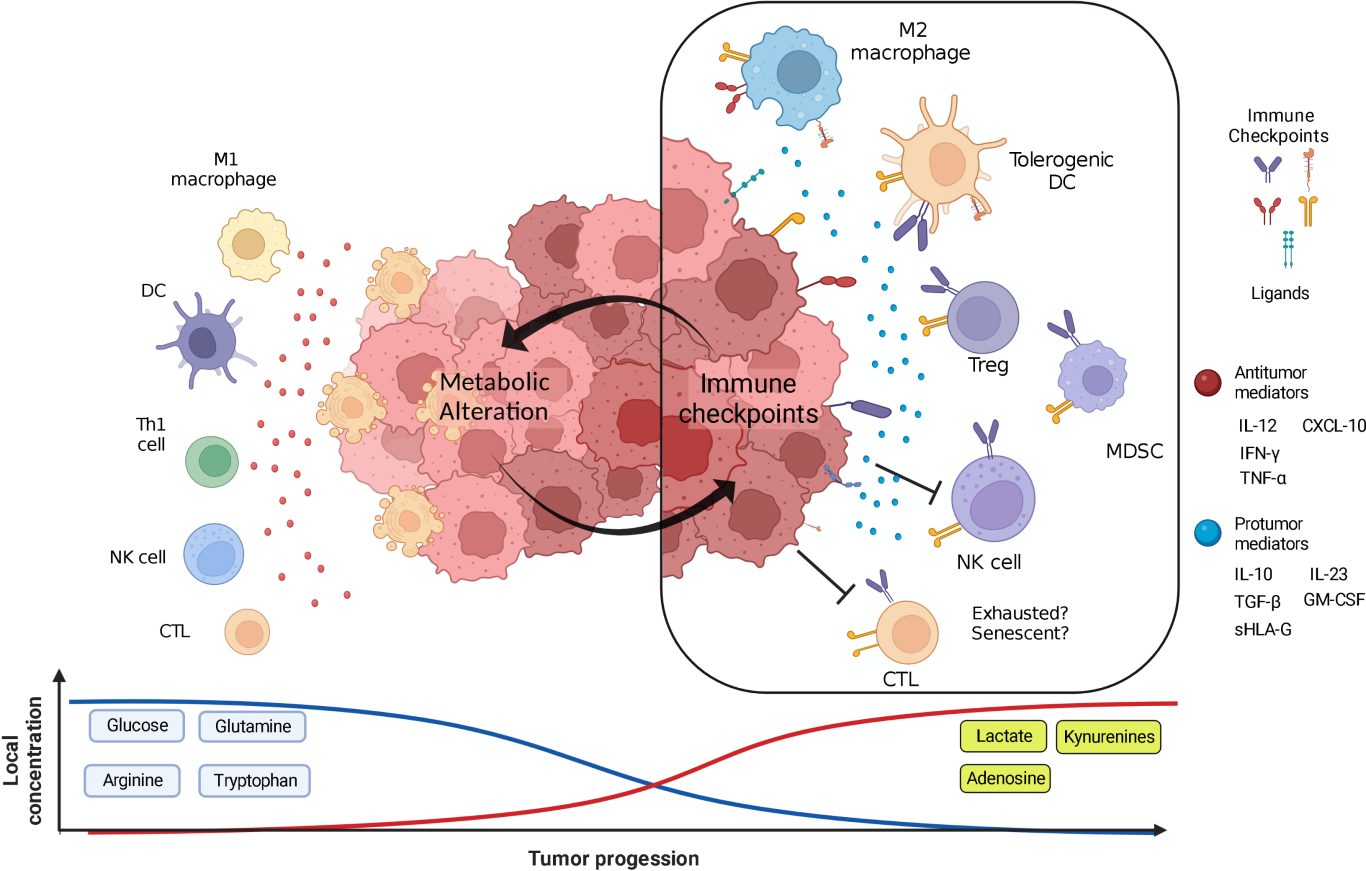
Metabolic shifting in the TME and its relationship with immune checkpoints. In early stages of tumor development, tumor and immune cells are immersed in a milieu enriched in nutrients such as glucose and amino acids. High nutrient availability allows antitumor immune cells to exert their effector functions, such as the production of soluble mediators against immunogenic and susceptible tumor cells. However, some tumor clones are able to resist the attack of immune cells and continue their uncontrolled proliferation. As a result of the sustained proliferation of tumor cells and the activation of the immune response, nutrients are gradually consumed, and metabolic by-products are accumulated in the TME. These metabolic alterations signal antitumor immune cells to end their effector mechanisms, as well as promote the recruitment and activation of immune cells with tumor-promoting phenotypes. Moreover, metabolic alterations establish a positive feedback loop with the expression of immune checkpoints and their ligands that strengthens the immunosuppressive state at the TME. See the text for detailed information. CTL=Cytotoxic T Lymphocyte, DC= Dendritic Cell, MDSC= Myeloid Derived Suppressor Cells, NK=Natural Killer cell, Th=T helper cell, Treg=Regulatory T cell, ⊥=Inhibition. Created with BioRender.com.

In cancer, the uncontrolled tumor cell proliferation and the activation of the antitumor immune response deplete local nutrient availability, combined with the by-products, resulting in a microenvironment similar to that of the resolution phase of the inflammation, perceived by immune cells as a signal to enter into a pro-resolution program. In this sense, despite tumor cells and antigens are maintained, immune cells in the TME terminate their effector programs or reprogram their activity to show regulatory function, promoting tumor progression. Moreover, the imbalance in nutrient availability and the accumulation of by-products induce the expression of immune checkpoints (ICs) and their ligands. Furthermore, the overexpression of ICs is linked to exacerbated metabolic alterations, which results in a positive feedback loop that strengthens the regulatory role of tumor-infiltrating immune cells. See **Figure 1**.

Multiple gaps remain to be elucidated. For instance, whether metabolic alterations are sufficient to drive exhaustion and senescence on T cells and if this relationship can be targeted by the combination of metabolic inhibitors and ICIs to restore the antitumor functions of TILs. Similarly, little is known about the effect of systemic metabolic alterations on the functions and phenotype of tumor-infiltrating immune cells. For this reason, it is necessary to deepen the understanding of the role that local and systemic metabolic alterations play on the functions of immune cells, as well as their crosstalk with ICs in the TME. Forthcoming knowledge derived from these aspects will aid the development of more efficient therapeutic strategies that improve the outcome of cancer patients.

## Author contributions

Conceptualization and design of the entire manuscript and original draft: JB-L, MM-M, MP-M., JL-G, DA-C, and MM-M wrote the participation of the immune response in tumor development. JB-L, JL-G, and MP-M wrote the metabolic reprogramming in the TME section. DA-C, RC-D, JB-L, and MM-M wrote the metabolic reprogramming drives the expression of ICs. JL-G and MM-M wrote the immune checkpoints feedback the metabolic alterations section. DA-C, MG-V, and RC-D wrote the targeting the crosstalk section. References were collected and incorporated in text by JB-L, MP-M, and MG-V. **Figure 1** was designed by JB-L, MM-M, and MP-M. **Tables 1** and **2**were designed by JB-L, MM-M, and JL-G. All authors contributed to the article and approved the submitted version.
